# Exploring the Relation Between Interstitial Lung Diseases and Chronic Periodontitis: A Systematic Review

**DOI:** 10.7759/cureus.53157

**Published:** 2024-01-29

**Authors:** Prerna S Hirkane, Umesh P Verma, Ajay K Verma, Pooja Singh

**Affiliations:** 1 Periodontology, King George's Medical University, Lucknow, IND; 2 Respiratory Medicine, King George's Medical University, Lucknow, IND

**Keywords:** antioxidants, smoking, genetic association, microbes, biomarkers, chronic periodontitis, interstitial lung diseases

## Abstract

The objective of this systematic review is to determine the association between interstitial lung diseases and chronic periodontitis from various aspects such as microbial, biomarker, genetic, and environmental levels. A systematic review was carried out from 2000 to 2021 following Preferred Reporting Items for Systematic Reviews and Meta-Analyses (PRISMA) recommendations including studies searched in PubMed-Medline, Google Scholar, and Cochrane databases. A total of more than 100 articles were obtained in the initial screening process. Out of these 42 studies fulfilled the inclusion criteria and were included in the study. According to the extracted data, there is mounting evidence suggesting the association between these two diseases. Our systematic review raises the prospect of a connection between chronic periodontitis and interstitial lung diseases, within the limitations of the studies we included.

## Introduction and background

Periodontal diseases are intricate, diverse chronic inflammatory infections that affect the bone and soft tissue supporting the teeth. The main etiological factors that lead to the degradation of supportive structures are microorganisms and their products in the plaque biofilm. The supposition that periodontal disorders may have an impact on certain systemic diseases has been supported by several studies published in the past decade such as cardiovascular diseases, diabetes mellitus, as well as multiple respiratory diseases. According to one of the theories, bacteria from plaque biofilm penetrate the bloodstream and induce inflammation and infection at a different location [1]. Growing evidence connects the microbiome to a range of lung diseases, and dysbiosis in the oral microbiome promotes periodontal disease. It has been documented that chronic periodontitis is linked to diminished respiratory capacity. One of the biological explanations for this association is systemic inflammation. The reduced respiratory function is often due to various lung conditions including chronic obstructive pulmonary disease (COPD), pneumonia, asthma, lung cancer, and interstitial lung diseases (ILD) [2]. ILDs represent a heterogeneous group of diseases characterized by chronic inflammation which results in scarring of the lungs. Despite the advancements in the treatment of ILDs, diagnosing a phenotype is challenging. Various biomarkers have increased the understanding of ILD and now started to contribute to ILD patient diagnosis, prognosis, and treatment.

The traditional clinical examination in large-scale research is time-consuming and costly. Therefore, other approaches, such as salivary diagnostics, have been suggested due to numerous inflammatory biomarkers, and elevated levels of periodontal pathogens have been detected in the saliva of periodontitis patients [3]. In the last decade, there has been a resurgence of interest in the potential effects of periodontitis on the cellular and molecular components of peripheral blood, including potential alterations in plasma proteins, such as acute phase proteins, immunoglobulins, and immunological mediators [4]. Most interestingly, the majority of the reviewed markers, microbes, genetic studies, and biochemical studies are associated with various lung conditions including ILDs.

## Review

Methodology

Search Strategy

A total of 42 studies were selected on the basis of inclusion criteria from the electronic databases PubMed-Medline, Google Scholar, and Cochrane. Some of the keywords used as a tactic in the article search were interstitial lung diseases, chronic periodontitis, biomarkers, microbes related to interstitial lung diseases and chronic periodontitis, genetic association, smoking, and antioxidants (Table 1).

**Table 1:** Search strategy of the study.

Database	Strategy
PubMed	Interstitial Lung Disease [All] OR idiopathic pulmonary fibrosis OR Sarcoidosis. Chronic periodontitis, biomarkers [All], microbes, genetic association, smoking, antioxidants.
Google	Interstitial lung Diseases [All] or Idiopathic pulmonary fibrosis, Periodontitis, Microorganism, Genes, Environmental factors OR Smoking, Antioxidants.
Google Scholar	Interstitial Lung Disease OR idiopathic pulmonary fibrosis OR Sarcoidosis. Chronic periodontitis, biomarkers, microbes, genetic association, smoking, antioxidants
Cochrane	Interstitial Lung Disease OR idiopathic pulmonary fibrosis, Chronic periodontitis, biomarkers, microbes, genetic association, Smoking.

Data Extraction

**Figure 1:**
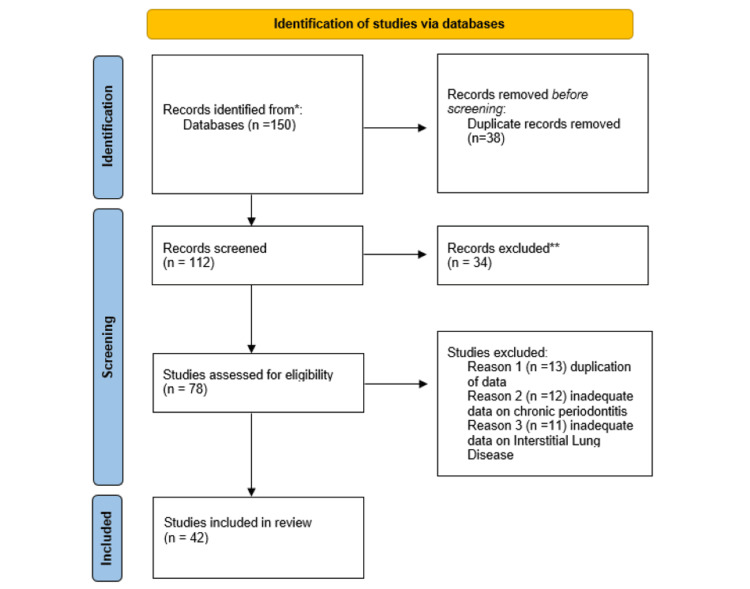
PRISMA flow diagram for study selection. PRISMA: Preferred Reporting Items for Systematic Reviews and Meta-Analyses.

This systematic review was carried out based on the Preferred Reporting Items for Systematic Review and Meta-Analyses (PRISMA) guidelines. The titles and abstracts of studies were first screened independently. For the studies satisfying the inclusion criteria, but with insufficient data in the abstract, full-text data were reviewed. Then, the shortlisted articles were subjected to a full-text review process to further narrow down to studies. For all the included studies, a validity assessment was done, and duplicates were removed at this stage. The steps involved in the search strategy have been shown in the PRISMA flowchart (Figure 1).

Criteria for inclusion of study in this review: All the studies were published in the English language within 20 years. These are categorized on the basis of disease, microbes, genetic association, environmental factors like smoking, and the impact of antioxidants and the specific biomarker of interest found among chronic periodontitis and ILDs.

Evaluation of methodological quality of included study: Out of 42 studies included in this systematic review, 15 are case-control studies, 14 are review-based studies, 5 are in-vitro experimental studies, 2 are clinical trials, 3 are cross-sectional, and 3 are cohort studies.

Results

Studies suggested that the microbiome may be a therapeutic target, a disease diagnostic, or an explanation for the development of chronic periodontitis and ILD (Table 2).

**Table 2:** Microorganisms associated studies of ILD and CP. IPF: Idiopathic pulmonary fibrosis; ILD: Interstitial lung disease; CP: Chronic periodontitis; rRNA: Ribosomal ribonucleic acid.

S. No.	Diseases	Microbes	References
1	Idiopathic pulmonary fibrosis	A faster-progressing disease was linked to Streptococcus or Staphylococcus species above a particular threshold.	Han MK et al., 2014 [5]
		Higher load of Streptococcus, Neisseria, and Veillonella genera in IPF as compared to healthy individuals.	Richter AG et al., 2015 [6]
		In IPF patients' bronchoalveolar lavage fluid (BALF), Prevotella and Staphylococcus species are shown to be the most common operational taxonomic units.	Huang Y et al., 2016 [7]
2	Sarcoidosis	Using 16S rRNA sequencing, it was discovered that Fusobacterium was more prevalent in sarcoidosis samples than in samples from healthy controls.	Zimmermann A et al., 2017 [8]
		Prevotellaceae, Streptococcaceae, and Acidaminococcaceae species found in abundance in sarcoidosis.	Garzoni C et al., 2013 [9]
		Periodontopathic taxa including Treponema, Prevotella, and Porphyromonas found in sarcoidosis.	Scher JU et al., 2016 [10]
3	Chronic periodontitis	Fusobacterium sp. (27.60%), Bacteroides fragilis (21.84%), Porphyromonas sp. (24.48%), Prevotella intermedia (9.20%).	Mombelli A, 2018 [11]

A biomarker is a measure of common biological processes, pathogenic processes, or responses to exposure or interventions. Biomarkers can be seen in saliva, gingival crevicular fluid (GCF), peripheral blood, airway, and lung parenchyma and may aid in the diagnosis, prognosis, and response to treatment of ILD and chronic periodontitis. Osteopontin, periostin, interleukin, matrix metalloproteinases (MMPs), and cathepsin-K have shown significant association between these two chronic inflammatory diseases. Osteopontin is a glycosylated non-collagenous, calcium-binding, phosphoprotein, abundantly present in numerous cells including osteoclasts, activated T cells, and activated macrophages. It has been discovered to play crucial roles in controlling biological functions such as tissue remodeling, immune regulation, and bone mineralization. Considering this, it has been stated that osteopontin may be crucial in the development of chronic inflammatory disorders (Table 3).

**Table 3:** Osteopontin in ILD and CP. ILD: Interstitial lung diseases; CP: Chronic periodontitis; OPN: Osteopontin.

S. No.	Disease	Osteopontin	Reference
1	ILD	Patients with ILD were found to have higher serum concentrations of OPN, which could help in diagnosis.	Goyal M et al., 2021 [12]
2	CP	The gingival crevicular fluid from areas with periodontal damage had the highest levels of OPN; however, periodontal therapy caused those levels to decrease.	Sharma CG et al., 2007 [13]

Periostin is a matricellular protein that modifies integrin-mediated cell function. There is growing evidence that periostin expression has a role in the healing process as well as other pathophysiological states of fibrosis which is present in both ILD and chronic periodontitis (Table 4).

**Table 4:** Periostin in ILD and CP. IPF: Idiopathic pulmonary fibrosis; IIP: Idiopathic interstitial pneumonia; PDL: Periodontal ligament; ILD: Interstitial lung disease; CP: Chronic periodontitis.

S. No.	Disease	Periostin	References
1	IPF	In patients with IPF, periostin is discovered at higher levels in the circulation and is substantially expressed in the lungs.	Naik PK et al., 2013 [14]
		Patients with IPF have serum periostin levels that are noticeably greater than those of patients with other IIPs, and these levels are inversely linked with pulmonary function.	Okamoto M et al., 2019 [15]
		Periostin is a significant contributor to a number of lung pathogenic pathways and may offer a helpful biomarker of clinical development in IPF.	O’Dweyr DN et al., 2017 [16]
2	CP	With the course and severity of periodontal disease, the levels of periostin in gingival crevicular fluid declined correspondingly, and they were inversely linked with the clinical parameters.	Bali U et al., 2015 [17]
		Periostin is thought to be essential for controlling PDL homeostasis.	Padial-Molina M et al., 2015 [18]

Interleukins (ILs) are a class of cytokines with immunoregulatory properties that are produced by a wide range of cells, including neutrophils, mast cells, macrophages, and T lymphocytes. Interleukins have a significant role in inflammatory diseases which also include ILD and chronic periodontitis (Table 5).

**Table 5:** Interleukins in ILD and CP. IL: Interleukin; IPF: Idiopathic pulmonary fibrosis; GCF: Gingival crevicular fluid; IFN: Interferon; ILD: Interstitial lung disease; CP: Chronic periodontitis.

S. No.	Disease	Interleukin type	Description	References
1	ILD	IL-1B	IPF patients had greater serum levels of IL-1, IL-2, IL-10, and IL-12 compared to healthy people.	Wilson, MS et al., 2010 [19]
		IL-6	In individuals with ILD, a high blood IL-6 level is a helpful biomarker for predicting acute exacerbation and a poor prognosis.	Lee JH et al., 2021 [20]
		IL-4	In pulmonary fibrosis, IL-4 can have functions like it may cause lung fibroblasts to express the collagen gene and encourage fibroblast differentiation.	Saito A et al., 2003 [21]
2	CP	IL-1B	IL-1 is crucial for periodontal tissues because of its ability to reduce bone growth and increase bone resorption.	Gamonal J et al., 2010 [22]
		IL-4	Compared to healthy gingival samples, patients with chronic periodontitis had significantly lower levels of IL4 and IFN.	Behfarnia P et al., 2010 [23]
		IL-6	IL-6 detected in the GCF of periodontitis patients enhances the synthesis of collagen.	Sachdeva S et al., 2020 [24]

MMPs, a class of zinc-dependent endopeptidases are required for the breakdown of extracellular matrix. The expression of MMPs and their biological inhibitors is closely regulated in the lung, and there is a noticeable increase during lung development, tissue injury, and host defense. Any imbalance between MMPs and tissue inhibitors starts the degradation of the collagens in the gums, which causes chronic periodontitis (Table 6).

**Table 6:** Matrix metalloproteinases in ILD and CP. MMP: Matrix metalloproteinase; TIMP: Tissue inhibitor of metalloproteinases; IPF: Idiopathic pulmonary fibrosis; ILD: Interstitial lung disease; CP: Chronic periodontitis.

S. No.	Disease	Matrix metalloproteinase	References
1	ILD	MMP-2 and MMP-7 concentrations were found to be higher among IPF patients.	Bauer Y et al., 2017[25]
		MMP-8 is higher in patients with IPF.	Craig VJ et al., 2014 [26]
		Patients with IPF have been shown to have elevated MMP-13 expression in their lungs.	Nkyimbeng T et al., 2013 [27]
2	CP	It has been shown that MMP-2 is crucial for the deterioration or turnover of soft connective tissue in chronic periodontitis.	Pattamapun K et al., 2003 [28]
		The protective TIMP-1 barrier may be breached by an activation cascade involving MMP-13 and -9.	Rios MH, et al., 2009 [29]
		Elevated concentrations of MMP-8 in individuals with chronic periodontitis.	Gupta N et al., 2015[30]

Cathepsin-K, a cysteine protease that is abundantly expressed, is important for both bone remodeling and cartilage degradation. Because this enzyme is mostly generated from osteoclasts, it is utilized as a well-known indicator of osteoclast activity (Table 7).

**Table 7:** Cathepsin-K in ILD and CP. GCF: Gingival crevicular fluid; ILD: Interstitial lung disease; CP: Chronic periodontitis.

S. No.	Disease	Cathepsin	References
1	ILD	In primary cell cultures, activation of human pulmonary fibroblasts increased cathepsin-K activity and intracellular collagenolytic activity.	Buhling F et al., 2004 [31]
2	CP	GCF from patients with periodontitis had a higher cathepsin-K level.	Mogi M et al., 2007 [32]

Table 8 displays the role of miR-21, miR-155, and miR-101-3p, miR-142-3p in chronic periodontitis and ILDs.

**Table 8:** Genomic studies associated with ILD and CP. LPS: Lipopolysaccharides; ILD: Interstitial lung diseases; CP: Chronic periodontitis; miR: MicroRNA.

S. No.	Disease	Genes	References
1	ILD	The expression of miR-21 was found to be markedly altered, and the upregulation of miR-21 was consistent with its expression level in IPF tissue.	Ping Li et al., 2014 [33]
		Radiological features of IPF and forced vital capacity (FVC) were linked with altered expression levels of miR-21, miR-155, and miR-101-3p.	
		It is hypothesized that miR-21 contributes to both inflammation and fibrosis because it is elevated during the inflammatory and fibrotic phases.	Blanca Ortiz-Quintero et al., 2020 [34]
		Elevated levels of miR-142-3p may cause a change in the equilibrium between mesenchymal cell proliferation and differentiation, a characteristic seen throughout the development of IPF.	Njock M et al., 2019 [35]
2	CP	MiR-21 is involved in periodontitis as evidenced by increased expression of the gene in periodontitis patients, ligated animals, and Porphyromonas gingivalis LPS-exposed cells.	Zhou W et al., 2018 [36]
		miR-142-3p is found to be associated with inflammation and LPS exposure.	Schmalz G et al., 2016 [37]
		By increasing the expression of certain cytokines, miR-155 is linked to important immune system regulators and is regarded as a sensitive biomarker for periodontitis.	Cuevas-González MV et al., 2021 [38]

Tables 9-10 depict the environmental studies such as the impact of smoking and antioxidants associated with chronic periodontitis and ILDs.

**Table 9:** Smoking-associated studies of ILD and CP. ILD: Interstitial lung disease; CP: Chronic periodontitis.

S. No.	Disease	Smoking	References
1	ILD	In recent years, the relationship between smoking and the pathophysiology of ILDs has changed. Smoking is increasingly recognized as a significant cofactor for the onset of ILDs.	Margaritopoulos GA et al., 2015 [39]
		The range of ILDs linked to smoking is greater than is typically recognized, and multiple kinds frequently coexist.	Attilli AK et al., 2008[40]
2	CP	Smokers are considered to be more prone to periodontal disorders and more likely to contract Porphyromonas gingivalis than non-smokers.	Buduneli N, 2021 [41]

**Table 10:** Antioxidant-associated studies of ILD and CP. IPF: Idiopathic pulmonary fibrosis; ILD: Interstitial lung disease; CP: Chronic periodontitis.

S. No.	Disease	Antioxidants	References
1	ILD	The literature is replete with examples of a wide variety of various antioxidants attenuating fibroproliferative processes.	Day BJ, 2008 [42]
		IPF and other diffuse lung disorders can advance as a result of elevated oxidant levels and lowered antioxidant defense.	Bargagli E et al., 2009 [43]
2	CP	Total antioxidant capacity was noticeably decreased in serum and plasma samples from people with periodontitis.	Chapple IL et al., 2007 [44]
		Numerous dietary elements that can act as antioxidants have the ability to enhance periodontal health and repair.	Najeeb S et al., 2016 [45]

Smoking is an extremely common and addictive behavior and because of the many chemicals it contains, it is well known for having harmful effects. The role of smoking in many human diseases, such as COPD, ILDs, and also in periodontal diseases has been defined over the years (Table 9). An imbalance between oxidants and antioxidants causes oxidative stress, which can have an impact on proteins, lipids, DNA, and carbohydrates. Antioxidants are substances that lower steady-state reactive oxygen species concentrations and defend cellular macromolecules from oxidative modification (Table 10).

Discussion

Poor oral health has systemic health consequences and the dental health of patients with lung disease is overall severely underappreciated. To the best of our knowledge studies have included respiratory diseases such as COPD, asthma, and bronchiectasis but very few studies have shown the least association of periodontal diseases and ILDS. Conventional clinical diagnostic criteria are insufficient to provide the relationship between the two diseases. Han MK et al., in 2014, extracted DNA from 55 samples of idiopathic pulmonary fibrosis (IPF) patients' bronchoalveolar lavage fluid (BALF) [5]. The preliminary findings of their study imply that the presence of particular *Staphylococcus *and *Streptococcus *genus members is related to the advancement of IPF. In a study by Huang Y et al. in 2017, combined information from BALF concluded that microbes such as *Streptococcus* and *Prevotella* species interact with the host immune in patients with IPF [7]. *Fusobacterium* species were associated with sarcoidosis and chronic periodontitis as well according to the studies done by Zimmerman A et al. in 2017 and Mombelli A in 2018 respectively [8,11].

Mombelli A reevaluates periodontal therapy tactics from the standpoint that the disease is a result of microbial colonization of the periodontal pocket environment [11]. *Fusobacterium*, *Porphyromonas gingivalis*, *Prevotella, *and *Aggregatibacter actinomycetemcomitans* are the common colonizers in chronic periodontitis. The microflora in chronic periodontitis patients and lung microbiota in ILD such as *Streptococcus *and *Staphylococcus* species as well as the *Prevotella *family may construct the link between two diseases.

In 52 patients of ILD, serum osteopontin levels were calculated using an enzyme-linked immunosorbent test by Goyal M et al. in 2021 [12]. Patients with ILD were found to have higher serum concentrations of osteopontin, which could help in diagnosis. Similarly, Sharma CG et al. in their study in 2007 included 30 subjects, using an enzyme immunoassay, samples of plasma and gingival crevicular fluid were measured for osteopontin [13]. GCF and plasma osteopontin levels showed a positive correlation in all of the groups. The periostin levels in gingival crevicular fluid decreased proportionally with the progression and severity of periodontal disease, as stated by Balli U et al. in 2015 [17]. In 2013, Naik PK et al. analyzed the expression of periostin with real-time quantitative reverse transcription-polymerase chain reaction (qRT-PCR) in a group of 54 IPFand came to the conclusion that plasma periostin may be a helpful biomarker for predicting early disease progression of IPF [14]. Hence, a nexus between the two diseases may exist between osteopontin and variations in periostin levels in chronic periodontitis and ILDs.

Wilson MS et al. in 2010 discussed the role of IL-1β and IL-17 in IPF and came to the conclusion that individuals with IPF had higher IL-1β levels in their BALF [19]. Likewise, Gamonal J et al., in 2000, conducted research; 12 patients with chronic periodontitis had their GCF and biopsies taken, and an enzyme-linked immunosorbent assay (ELISA) was used to evaluate the cytokine levels [22]. The findings demonstrated that active sites of Periodontitis had higher levels of IL-1β than inactive sites. Retrospective clinical data analysis was done on 83 individuals who received an ILD diagnosis between 2016 and 2019 by Lee JH et al. [20]. As a result, it can be said that a high blood IL-6 level is a helpful biomarker for predicting acute exacerbation and a bad prognosis in individuals with ILD. Saito A et al., 2003, in their in-vitro study, summarized that IL-4 in pulmonary fibrosis encourages the differentiation of fibroblasts into myofibroblasts and enhances the gene production of collagen in lung fibroblasts [21].

The level of the specific antibody in IFN, IL-4, and IL-17 cytokines was quantified using the ELISA method and the results were compared by Behfarnia P et al. in 2010 [23]. In comparison to healthy gingival samples, patients with chronic periodontitis had significantly lower levels of IL-4 and IFN. In the context of ILD and chronic periodontitis, MMPs have been implicated in the pathogenesis of the disease. Bauer Y et al., 2017 collected ILD patients’ serum samples which were utilized to measure (MMP-7), evaluate their prognostic potential, and track changes as the disease progressed [25]. MMP-7 showed the potential to be a reliable predictor of lung function decline and disease progression. 

By employing ELISAs, western blotting, and quantitative polymerase chain reaction (qPCR) to quantify MMP-8 levels in blood and lung samples from IPF patients versus controls, Craig VJ et al., 2014, investigated MMP-8 plasma levels in 73 IPF patients and concluded that blood and lung MMP8 level is increased in IPF [26]. In the prospective investigation done by Rios MH et al. 2009, the MMP-13 activity in GCF samples from chronic periodontitis patients and healthy controls was checked [29]. Gelatine zymography and densitometric analysis were used to evaluate diseased gingival explants and draws the conclusion that the deterioration of both soft and hard supporting tissues as well as the activation of proMMP-9 during the development of chronic periodontitis may be related to MMP-13. Periodontal ligament (PDL) cell cultures were exposed to *Porphyromonas gingivalis* supernatant for 48 hours, and gelatin zymography was used to measure the degree of MMP-2 activation by Pattamapun K et al. in 2003 [28]. MMP-2 was only severely activated in the chronic periodontitis group. Increased concentrations of MMP-2, MMP-7, and circulating MMP-8 have been found in patients with IPF while connective tissue deterioration in chronic periodontitis has been shown to be significantly influenced by these MMPs. Thus, from the above research, MMPs could be a strong link between ILDs and chronic periodontitis.

In a study by Buhling F et al., 2004, diagnostic open lung biopsies were used to obtain tissue samples from patients with and without lung fibrosis (fibrotic samples) [31]. By using qRT-PCR, they evaluated the cathepsin-K mRNA levels. In general, patients with lung fibrosis had cathepsin-K mRNA levels that were almost three times higher. Mogi M et al., 2007, reported the GCF sample of 66 patients with chronic periodontitis [32]. ELISA kit was utilized to ascertain the cathepsin-K total content. According to the studies mentioned above, cathepsin-K may act as an association between two diseases.

The data collected in regard to the genomic and environmental association between chronic periodontitis and ILD is discerned to be supportive. Ping Li et al., 2014, performed microRNA analysis and data was confirmed using qRT-PCR [33]. The results revealed that the expression of miR-21, miR-155, and miR 101-3P had significantly changed in the serum of IPF patients. Seven patients with periodontitis provided PDL tissues in the investigation done by Ortiz-Quintero et al., 2020 [34]. Using Trizol, total RNAs were extracted from the tissues. The findings of the study demonstrated that miR-21 reduced the inflammatory response to *Porphyromonas gingivalis* lipopolysaccharide (LPS). In an investigation done by Njock M et al., 2019, exosomes were separated from the generated sputum supernatant using a conventional ultracentrifugation procedure [35]. In the results, they discussed that MiR-142-3p may alter the balance between mesenchymal cell proliferation and differentiation, which is a feature seen throughout the progression of IPF. Schmalz G et al., 2016, in their review, concluded that, in chronic periodontitis, miR-142-3p is reported to be increased and linked to inflammation and LPS exposure [37]. The altered expression of miR-21, miR-155, and miR-101 3p is significant in both diseases, and elevated levels of miR-142 3p in ILD and chronic periodontitis may cause a change in equilibrium and is related to inflammation.

Smoking and periodontal health relation are well documented in the literature while in recent years, smoking is considered as a significant co-factor for the outset of ILDs. According to Margaritopoulos GA et al., 2015, smoking is now thought to be the primary pathogenetic factor for respiratory bronchiolitis (RB)-ILD; however, for the development of other ILDs like IPF and rheumatoid arthritis (RA)-ILD, smoking is likely to work in concert with genetic and environmental variables [39]. While in context to chronic periodontitis according to Buduneli N, 2021, smokers are thought to be more susceptible to periodontal conditions and more likely to get *Porphyromonas gingivalis* than non-smokers [41]. Reduced plasma antioxidant levels in chronic periodontitis, as well as ILD, could be viewed as a common factor between these two disorders.

## Conclusions

Multiple research has shown the relationship between reduced respiratory capacity and periodontitis. Our systematic review suggests the potential association between chronic periodontitis and interstitial lung diseases. Microbiological studies create a strong link between two diseases with some common microbial species such as *Streptococcus* and *Staphylococcus *species as well as *Prevotella *species, while biomarker-related studies form a nexus of hypothesis.

Inflammatory proteins such as osteopontin, periostin levels as well as variety of cytokine measures indicate that an interrelation exists between both chronic diseases. The high correlation between the two diseases is also attributed to the prevalence of certain matrix metalloproteinases such as MMP-2, MMP-7, MMP-8, MMP-9, MMP-13. Genetic and environmental factors such as smoking and imbalance of oxidants and antioxidants in the diseases, additionally contribute to stepping up the association. However, the evidence-based relation between diseases still needs further investigation for more conclusive results.
